# The “*Ensemble*”—A Group Music Therapy Treatment for Developing Preschool Children’s Social Skills

**DOI:** 10.3390/ijerph19159446

**Published:** 2022-08-02

**Authors:** Raya Blanky-Voronov, Avi Gilboa

**Affiliations:** Music Department, Bar-Ilan Univerisity, Ramat Gan 5290002, Israel; avi.gilboa@biu.ac.il

**Keywords:** social skills, music therapy treatments, mixed methods, children, group work, group dynamics

## Abstract

(1) Background: Several music therapy treatments have been developed to assist children with social skill deficiencies. They lack reference to emotions and their connection to social skills and they don’t deal with group dynamics and its impact on the group. We conducted a preliminary examination of the “*Ensemble*” treatment, which was developed to improve the social skills of children of various client populations, with the social deficiency originating from different sources; (2) Methods: 24 children in four groups went through the year-long “*Ensemble*” treatment. Observations of the sessions were analyzed quantitatively counting the occurrence of twelve typical socially oriented behaviors. Qualitative interviews were conducted with 24 mothers and 23 kindergarten teachers (KTs) before and after the process; (3) Results: Quantitative results show that children in all four “*Ensemble*” groups, improved in nine out of twelve social skills. Qualitative results show that the improvement was evident also in the home environment (as reported by mothers) and in the kindergarten environment (as reported by KTs); (4) Conclusions: A comparison between the “*Ensemble*” and previous music therapy treatments indicates this treatment’s potential to help children with a wide variety of social skill deficiencies. Further investigation based on more rigorous research designs is recommended.

## 1. Introduction

The “*Ensemble*” is a group music therapy treatment for developing preschool children’s social skills. It is called “*Ensemble*”, as a metaphor to a musical ensemble where group members must learn to work together and be attentive to each other even if they have challenges doing so because their social skills are not yet developed. The term “social skills” denotes the abilities that enable appropriate social behavior, which is expressed in one’s ability to meet society’s expectations, foster social relationships with peers, adapt to the demands of reality, and make decisions in emotional, personal, social, and value contexts [1]. Social skills are expressed in different ways, such as taking initiative and making an effort to engage with others; being able to observe, listen, and perceive the subtleties of social situations; developing different conversation skills; cooperating with others and being able to receive help from others, and empathizing with others [2].

Social skills are fundamental to a child’s development [3,4] and it is therefore important to offer effective treatment that can help children with these types of deficiencies to improve and be able to communicate socially with their peers. This can be a complex task since various reasons may underlie the problem. First, children with physical disabilities such as hearing problems and visual impairments may have difficulty integrating socially and acquiring the required skills [4,5]. Second, motor and language developmental delays may hinder social development [6]. More severe developmental difficulties characterize children on the spectrum of communication disorders. These children may have significant difficulties forming interpersonal relationships in general and acquiring social skills in particular [7,8]. Third, children with sensory processing disorder and emotional difficulties may respond inappropriately to what the environment requires. Daily functioning is impaired, adapting to challenges is slow and partial, as is social adaptation [9]. Fourth, children with learning disabilities, including children with verbal disabilities, often fail to clarify their intentions and subsequently experience frustration and give up expressing their opinions. They might end up feeling that they are not understood, and this may hinder their social development [10]. Fifth, ADHD, which most often manifests as restlessness, low frustration tolerance, impulsivity, hyperactivity, and distraction, also affects the child’s social relationships [11,12]. In any of these possibilities, the result may be withdrawal from and avoidance of social interactions and consequently, lack of opportunities to acquire the social skills that are so important for development [13].

Music therapy has been found to effectively address such a lack of social skills and it has been used with various client populations with the social deficiency originating from different underlying sources. Montello and Coons [10], for instance, found that music therapy helped to reduce hostility levels in children with emotional, learning, and behavioral disorders, and Rickson and Watkins [12] found that music therapy increased the responsiveness to social cues and positive social behavior as well as encouraging interactions with peer groups in aggressive adolescent boys. Throughout the years, different treatments that aimed to promote children’s social skills have been developed. Improvisational Music Play (IMP), developed by Gunsberg [14], aims to encourage playfulness between regular and developmentally challenged children when integrated in a standard educational system, and to encourage long-lasting play between them. Play between the children is prompted in response to the therapist’s improvisation. This method spontaneously develops social skills, with the normal developing children serving as a role model for children with developmental delay. For example, when a developmentally challenged child sees a normal developing child playing a socio-dramatic game with another child, s/he may try to join the game and emulate the child’s way of playing.

The Levels System Approach (LSA), developed by Presti [15], aims to rehabilitate and integrate children with emotional difficulties into regular classrooms. This method is essentially behavioristic, and music is used to shape the children’s behavior. The advantage of the method is that it creates a gradual process that moves from the individual to the general. Moreover, there is a gradual increase in the degree of difficulty, which increases the children’s sense of success. This treatment helps children learn behavioral norms such as postponing gratifications, waiting for the turn, emotional regulation, consideration for the other and strengthening social relationships. For example, when a child has to wait his or her turn to play a musical instrument, s/he needs to learn to be aware that other children are currently playing, and that it is important to let them finish playing.

Musical Interaction Therapy (MIT) was developed by Wimpory et al. [8] in an attempt to assist autistic children to cope with social difficulties. The method focuses on the parents’ behaviors and making them more predictable for the child, thus helping to improve social interactions between the parent and child. The method’s advantage is that it is structured and has evaluation criteria. During the treatment, it is possible to see what stage the child is at and what the aim should be. The main purpose of the treatment is to increase active social participation, through social initiative and eye contact. It includes games that are based on physical contact and imitation of the child’s behavior, thus enabling joint activity between the child and the parent and helping the child to develop symbolic play and social communication. For example, while the child is playing with his or her parent, the parent can encourage eye contact and interaction. Over time, the child can learn to apply these skills among peers.

Music Therapy Social Skills: Assessment and Documentation Manual (MTSSA), developed by Rook et al. [16]. This method is designed for children with developmental delay who attend regular schools. It assesses their social skills for future development strategies. The purpose of this treatment is to encourage spontaneous playfulness and interaction among the children. Learning is imitated when more communicatively advanced children help children with social difficulties. The method’s advantage is that it is structured and focuses on the participation of parents and teachers in the therapeutic process. The method is suitable for children of different ages and with different disabilities. This treatment helps children to create interactions independently. For example, a child who has difficulty playing a particular game receives help from another child in his or her group. This develops good communication and a general sense of belonging.

Although these treatments have important qualities, they were not systematically tested. In addition, they each have some drawbacks. IMP [14] lacks the element of a continuous long-lasting group process. The method is comprised of play sessions that do not occur consistently and it lacks an in-depth focus on emotions. There is no discussion of emotions that surface during an activity and ways to cope with them. Moreover, this method applies mainly to children with cognitive disabilities and is less suitable for children with other disabilities. The LSA, being behavioristic, lacks direct reference to emotions, emotional processing, and any other emotional guidance that can help children understand their disability. MIT focuses on the parent-child dyad, and it lacks group processes of any kind. MTSSA focuses on the evaluation process and does not offer ideas for further treatment. Clearly, a treatment that can encompass all needs and bridge the aforementioned gaps could be useful for music therapists working with children that need to develop their social skills.

To address this problem, I (The first author of this article) developed the “*Ensemble*” treatment, which integrates the advantages and avoids the disadvantages of previous treatments, and I have been using it with good results. We will first describe the treatment, its goals and underlying principles, and then report the findings of a study we conducted as part of my doctoral dissertation, to examine whether the treatment improved children’s social skills.

The “*Ensemble*” treatment is a method of structured work with small groups of 5–6-year-old children with social difficulties that stem from various underlying reasons. Sessions are weekly and conducted over the course of a school year (up to 35 weeks) in a room equipped with musical instruments and art supplies. Sessions are varied and include structured activities, improvisations, games, and short films. In addition to the sessions with the children, the music therapist meets with each of the children’s parents and with their kindergarten teachers at the beginning of the process, mid-term, and at the end of the process. 

The primary goal of this treatment is to develop and improve the children’s social skills. This is broken down into 6 sub-goals and 12 measurable behaviors: (a) *to strengthen verbal interactions* (i.e., initiating contact with a friend (1), answering a friend (2), initiating contact with the therapist (3), sharing (4)). This is achieved, for example, by asking children to tell each other about experiences they had during the week; (b) *to strengthen non-verbal interactions* (i.e., participating in group activities (5), making and maintaining eye contact (6)). This is achieved, for example, by a musical activity called “alone and together” in which each child chooses a musical instrument and in turn plays on it alone and the groups joins only after s/he have signaled that they can join. This activity is performed without words so that children need to learn to be very articulate about their intentions; (c) *to develop emotional awareness* (i.e., sharing emotions with others (7)). This is achieved by explicitly addressing emotions, from session 4 to session 10—focusing on a different emotion for each of these sessions. In a session that deals with sadness, for instance, a sad story is read and then discussed and shared. Then children choose an instrument to play something sad and then the group improvises on this theme together; (d) *to develop an awareness of others* (i.e., being considerate of a friend (8), helping a friend (9). This is achieved by initiating situations that require social awareness. For example, one child plays a certain rhythm on a drum and the others have to repeat him with accuracy; (e) *to learn strategies for solving social problems* (i.e., reaching a compromise (10), providing solutions to different social problems (11)). This is achieved as conflict spontaneously arises during sessions, and there is need to find ways to resolve them. For example, if two children want to play the same instrument, first priority is given to thinking of different ways to resolve the situation; and (f) *to improve self-control* (i.e., the ability to delay gratification (12)). This is achieved throughout the sessions whenever a child has to follow what the group is doing before doing what s/he initially want to do. For example, during a musical activity, the ability to imitate others patiently before playing a solo, or the ability to play softly to enable others to be heard, even though there is an urge to play loudly. 

To attain these goals, several principles are practiced: (1) Centrality of emotions: Children are encouraged to express their feelings and are assisted in understanding the emotional expressions of others in the group; (2) Gradual movement from individual work in the first sessions to pair and group work as the year progresses; (3) Clear structure: There is a planned order to the meetings and their content, and a consistent setting within every session; (4) Combination of art mediums: Use of music, art, and story activities, each of which adds its unique value and enables different avenues of expression for the children. Structurally, there are three important points: (1) The treatment duration is an entire school year, not less. This enables the emotional processes and social connections to develop; (2) Group size is optimally between five and seven children. A smaller group does not enable enough diversity, and divisions to subgroups are limited, while a larger group might be intimidating for some children with social disabilities; and (3) Communication with parents and kindergarten teachers provides their feedback and facilitates updates on progress or specific problems.

The aim of the present study is to conduct an initial examination of the “*Ensemble*” treatment, and to determine whether it has the potential of improving children’s social skills. This study focused on two main areas. First, it examined whether children improve their social skills within the confines of the group. Second, the study examined whether the children generalize the social skills acquired in the group and apply them in contexts other than the group. The research questions were:

1. Does the *“Ensemble*” treatment improve children’s social skills?
2. Is the impact of the “*Ensemble*” treatment apparent outside the confines of the group?

## 2. Methods

The study was approved by the Ethics Committee of the Music Department at Bar-Ilan University (Approval number: E.MUS 2015-2). Participants signed an informed consent form prior to the beginning of the study, and it was clarified that they could leave the study at any time without affecting their ability to stay in the treatment, and that their details were kept confidential. Participants were given information about the research objectives and the planned procedures. 

To answer the research questions, we conducted four *“Ensemble*” groups throughout a school year and applied mixed quantitative and qualitative methods [17]. Specifically, we used a *simultaneous design* applying quantitative and qualitative analyses on data that we collected in the same timeframe. Quantitative analysis was based on observations of video recordings of the sessions and counting key behaviors, and qualitative analyses were based on feedback received from the children’s mothers and teachers, attained through semi-structured interviews. Power analysis for *t*-test pairs with α = 0.05, power = 0.8, and medium to large effect size = 0.6, resulted with a *n* = 24 sample size.

### 2.1. Participants

Twenty-four children between the ages of 5 and 6 (one year prior to first grade) participated in the study. They were selected according to their kindergarten teacher’s assessment as experiencing challenges with social skills. All participants were from families from the working middle-class socio-economic status.

Twenty-four parents participated in the study, the more involved spouse of each of the children participating in the study. Although either fathers or mothers could participate, the mothers of all 24 children were the ones who chose to be involved in the study. All participants were from middle-class working socio-economic status. 

Twenty-three kindergarten teachers (KT) participated in the study, one KT per participating child, with one KT excluded from the study per the request of that child’s mother.

### 2.2. Tools

Observation: The sessions of all four “*Ensemble*” groups were videotaped. A wide-angle GoPro Hero camera was placed in a fixed position in the treatment room on a tripod, at an angle that covered the entire room. No person was required to activate the camera and the intimacy of the groups was thus maintained. 

Semi-structured interviews [18] were conducted separately with each of the mothers (i.e., 24 interviews) and the teachers (i.e., 23 interviews). The interview questions were prepared in advance to direct the participants to the topic being explored and to clarify important points [19]. Sample questions included “Can you tell me about the child’s social life? Does s/he have any friends? Does s/he meet with them? If so—what is the nature of these meetings? Do you think the child understands social situations and social codes? Can you give examples to substantiate yourself? Interviews were recorded on a Samsung Galaxy Note 3 and later transcribed by the researcher.

Treatment room: The study was conducted in a fully equipped music therapy room that was outfitted with musical instruments and art supplies that were made available in accordance with the treatment plan. 

### 2.3. Procedure

First, KTs recommended the children in their kindergarten who they thought needed to improve their social skills. After selecting 24 children (from 24 different kindergartens), the idea of the “*Ensemble*” treatment was explained to the parents in an intake session, and they were asked to provide assent for their children’s participation in the study. Parents could choose whether or not to participate in the music therapy sessions and/or in the research study. All parents agreed to participate in the music therapy sessions and in the research study, and signed informed consent forms. The exception to this was one parent who agreed to participate but requested that the child’s KT not participate in the study. The KTs of the children who were to participate in the study (except for the one whom the mother excluded) were contacted and asked to participate in the study. All the KTs agreed and signed informed consent forms as well. One-on-one interviews were conducted with the mothers and the KTs, in which initial acquaintances were made and interviewees had an opportunity to speak about the child. The children were then divided into four groups according to clinical considerations (e.g., the children’s personality and challenges, assuring that the groups were socially heterogeneous).

Groups were held as part of the afternoon treatment center which is operated by the Ministry of Education. Children who have been referred by kindergarten teachers or schools for individual or group treatment are eligible for this treatment center. Groups began in October 2012, one month after the school year began, and ended in May 2013, about one month before the school year ended. A total of 25 sessions were conducted. The mothers and KTs were interviewed again in person, mid-term and then at the end of the year. The interviews were recorded for subsequent analysis.

### 2.4. Data Analysis

Twelve out of 25 “*Ensemble*” sessions were sampled in order to provide a representation of all stages of the treatment and of different types of activities (primarily musical, music and art, verbal interactions, play, etc.). To answer the first research question (“Does the “*Ensemble*” treatment improve children’s social skills?”), a quantitative analysis of the children was conducted. Videos were observed by a research assistant who counted the frequency of social behaviors expressed by each of the participants during each session. In the coding of the first sessions, 10% of the videos were sampled and coded by the first author of this article and compared to the coding of the research assistant. A high inter-judge score was obtained (α = 0.95) and discrepancies in coding were negotiated to maintain high inter-judge reliability. Twelve social behaviors were counted, in accordance with the behaviors that were detailed in the description of the “*Ensemble*” treatment: initiating contact with a friend, answering a friend, initiating contact with the therapist, sharing, participating in group activities, making and maintaining eye contact, sharing emotions with others, being considerate of a friend, helping a friend, reaching a compromise, providing solutions to different social problems, the ability to delay gratification. The higher the number of behaviors per session, the better the participant was doing socially. To examine whether the number of behaviors improved by the end of the treatment we conducted paired *t*-tests to compared the average frequency of each of the 12 behaviors in the beginning vs. the end of the treatment. We first compared the first session to the last one and then the average of the first three sessions to the average of the three last sessions.

To answer the second research question (“Is the impact of the “*Ensemble*” treatment apparent outside the confines of the group?”), all interviews with the mothers and with the KTs were qualitatively analyzed. Smith and Osborn’s [18] interpretative-phenomenological analysis (IPA) framework was used to understand how the mothers and the teachers referred to the child’s needs, challenges, strengths, and whether s/he improved or not throughout the year. We based this analysis on the interviews that were conducted at the beginning and at the end of the process (24 × 2 mother interviews and 23 × 2 KT interviews). 

Research credibility was obtained in several ways: (1) Observation: The analysis was based on video footage, which helps to overcome memory biases. Interviews were also recorded and kept available for analysis and verification.; (2) Transcribed interviews were sent to the interviewees to verify that the verbatim were accurate; (3) An external observer was asked to examine the videos and add comments to enrich the findings; (4) To achieve triangulation, data was gathered using different sources (i.e., video recordings, interviews with mothers, and interviews with KTs).

## 3. Results

### 3.1. Research Question No. 1 (Quantitative Results)

**Figure 1:**
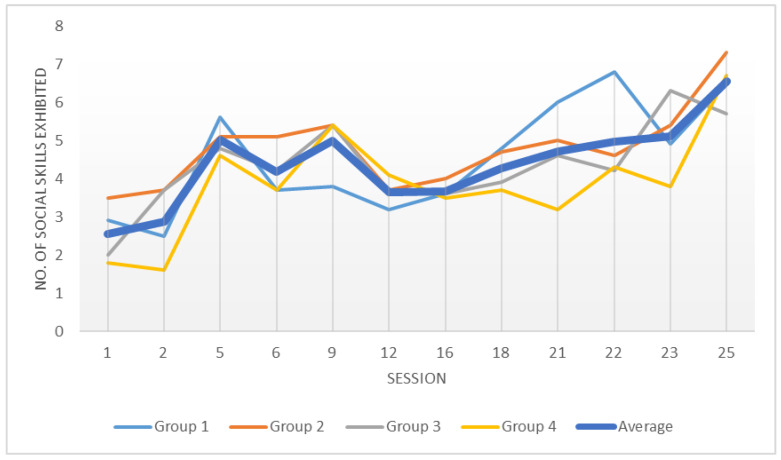
Distribution of average children’s behaviors by group.

To examine the first research question and to determine whether a change in the participants’ social abilities occurred throughout the “Ensemble” treatment, we plotted the average number of social skill behaviors exhibited by group participants from the first session to the last. As can be seen in Figure 1, a constant increase in social behaviors was evident in each of the four “Ensemble” groups separately, as well as when averaging participants of all groups together. A minor decease in social behavior in all groups could be noticed between sessions 9 and 12. This temporary regression could be explained by the fact that these sessions were dedicated to processing unpleasant emotions such as sadness, anger, and fear. Equipped with these emotional skills, participants could continue improving as the group process continued. 

To examine whether these differences were statistically significant, we conducted a paired *t*-test to compare the frequency of each of the social behaviors between the beginning of the process (the first session) and the end of the process (the last session). As can be seen in the middle three columns of Table 1, most social behaviors had significantly improved in the last session in comparison to the first session. The exception to this was “being considerate of a friend” and “helping a friend”, where no differences were recorded between the first and last sessions, and “providing solutions to social problems”, which significantly decreased from the first to the last session. 

To establish these finding and to exclude the possibility that the improvement only occurred in the final session, we repeated the statistical analyses, this time comparing the averages of the three first sessions with the three last ones. As can be seen in the three rightmost columns of Table 1, results stayed the same, with nine behaviors showing significant improvement, two measures showing no improvement, and one measure showing a significant decline.

### 3.2. Research Question No. 2 (Qualitative Results)

In order to examine the second research question and to determine whether the impact of the “Ensemble” treatment was apparent beyond the therapeutic environment, we qualitatively analyzed the interviews with the mothers and with the KTs, at the beginning and the end of the process. This analysis was done in several stages according to the interpretative-phenomenological analysis (IPA) framework [18]. First, we wanted to use the rich information that emerged during the initial interviews to get a sense of the children’s basic difficulties, according to the mothers and KTs. Then, based on a qualitative category analysis we found the following five types of difficulties (Note that these evaluations are in no way professional diagnoses. A professional diagnosis is typically conducted by a neurologist only when the child reaches the age of 6). 

Emotional difficulties pertaining to a problem in the child’s emotional conduct. For example, one of the mothers said “my son cries in response to every little thing and can barely handle any frustration. It’s hard for me to hear him cry!”. Another mother said “my daughter doesn’t share feelings and has frequent outbursts of rage. I’m really scared to invite her friends over because if she gets into such a mood she can become physically aggressive…”. Other examples included unregulated expression of emotion or lack of emotional expression.

Social difficulties pertained to the relationship between the child and his or her peer group. For instance, one of the mothers said “my son has a hard time connecting with his peers. When we go to the playground, kids play together, but he stays alone. It really hurts me to see him like this”. One of the KTs said about a child in her kindergarten, “he hardly ever initiates interaction with other children. He can play near other children, but will almost never initiate contact with them”.

Behavioral difficulties pertained to aggressive behaviors towards peers or defiant behaviors towards the teacher, the mother, or any other authority figure. For example, one of the KTs stated about a child in her kindergarten that “he has difficulty accepting authority and boundaries. He is physically aggressive towards other children in the kindergarten and sometimes he is verbally aggressive towards them. I’m afraid to take my eyes off him because he can hurt other children”. One of the mothers said about her child “every time my son loses a game, he becomes physically aggressive towards his siblings. Friends no longer want to come over and play with him”.

Speech difficulties pertained to problems in expressing, organizing, or understanding verbal communication with peers or with adults. For instance, one of the mothers said “my daughter often doesn’t understand me. I have to repeat the request several times in different ways so that she understands what I mean”. One of the KTs stated about a child in her kindergarten that “Leon has difficulty naming and retrieving words, and because of this it’s difficult for him to communicate with other children at the kindergarten. I need to be by his side and help him communicate with other children”.

Attention and concentration difficulties that typically manifest in excessive restlessness, daydreaming, inability to concentrate, etc. For example, the kindergarten teacher said, “Shai has a difficult time focusing his attention. He jumps from game to game and is unable to finish it. Eventually, he finds himself playing alone because the other kids continue with the game they started”. One of the mothers said “my daughter doesn’t rest for a moment. She isn’t even able to sit while eating. She gets up a lot of times during the meal to do countless things, but then she forgets why she got up in the first place”.

After substantiating these five types of difficulties, we examined which of them were reported for each of the children at the beginning and at the end of the process. We reviewed the interviews with the mothers to see which types of difficulties they reported in the first interview, and which difficulties they reported as persisting, regressing, or improving by the second interview. We repeated this process independently with the KT interviews. 

Table 2a–d summarize these reports, divided into four groups of children. The “<” symbol indicates an improvement in a reported difficulty from the beginning to the end of the process, “=” indicated no change, and “>” indicates a regression in that type of difficulty. Empty rubrics indicate that this type of difficulty was not reported for this child by either the mother or the KT. For instance, Roi (All names in this article are pseudonyms), who appears in Table 2a, was reported to have emotional and social difficulties both by his mother and his KT, and behavioral difficulties by his KT. The “<” signs in these cells indicate that by the end of the process, Roi’s mother and KT reported an improvement in all of these difficulties. He was not reported to experience speech difficulties or attention difficulties. Another example is Michael from Group 3, who was reported to experience social, behavioral, and attention difficulties, both by his mother and his KT. He improved with regard to his social difficulties as indicated by the “<” signs in both mother and KT columns, but did not improve with regard to his behavioral difficulties as indicated by the “=” in the respective cells. Attention-wise, the mother reported an improvement (“<”), while the KT reported no improvement (“=”). Neither his mother nor his KT reported speech difficulties.

In general, Table 2a–d show that mothers and KTs reported an improvement in most of the difficulties that the children experienced. The fact that an improvement was reported separately by the mothers and the KTs and that there was an improvement in different types of difficulties, indicates the effectiveness of the “Ensemble” treatment not only within the confines of the group sessions but also in the home and class environments.

## 4. Discussion

The aim of the study was to initially examine the “*Ensemble*” treatment and to determine whether it has the potential of improving children’s social skills, inside and outside the group setting. Results showed that social skills improved significantly from the beginning of the group process to the end, and that this improvement was consistent within each of the four groups participating in the study. Results also showed that children improved their social skills outside the group setting as reported by their mothers and their KTs. All in all, results indicate the “*Ensemble*” treatment’s potential to improve children’s social skills and that the improvement extended beyond the group environment to the child’s day-to-day interactions.

It is noteworthy that all four groups in this study had a heterogeneous composition of participants. As can be seen in Table 2, children in each group had various types of difficulties, the gender distribution differed from group to group, and in some groups there were participants who consistently challenged the therapists’ authority. Yet, findings indicate an improvement among the children in all groups, regardless of the groups’ diversity. This strengthens the validity of the “*Ensemble*” group, and it confirms the premise that “*Ensemble*” is an effective treatment for a spectrum of difficulties.

In contrast to the findings’ general trend indicating an improvement in social skills, “providing solutions to social problems” decreased. A possible explanation for this is that within the groups the children felt contained compared to the routine at kindergarten, and therefore felt more relaxed and did not need to fight for their place, which led to fewer social problems that needed to be resolved. Interestingly, in the other settings—kindergarten and home—there was evidence of an increased frequency of this behavior. It is possible, therefore, that the existence of the group had an emotional appeal and boosted self-confidence among the children so they could provide a successful solution to a social difficulty outside the treatment group. The frequency of two behaviors did not increase following treatment: being considerate of a friend and helping a friend. It is important to pursue further studies to refine the treatment and to understand how these important social behaviors can be enhanced as well.

A comparison between the “*Ensemble*” treatment and other music therapy treatments that focus on social skills can point to the advantages and disadvantages of the various treatments as well as the uniqueness of the “*Ensemble*” treatment. Table 3 compares between the main treatments that pertain to social skills (see a description of these treatments in the literature review) according to various parameters, including the purpose of the treatment, its level of construction, target population, art mediums used, reference to treatment process, to emotions, and to group dynamics, reference to parents and teachers, group size, and research on the treatment.

A summary of Table 3 indicates that “*Ensemble*” is a unique treatment with several advantages compared to the other treatments. It enables work on a wide range of social skills. It has a good balance between structure that provides confidence and spontaneity while allowing for interactions and free play. The treatment encompasses diverse target populations over a wide age range. It incorporates other types of mediums that provide children with diverse modes of expression. It is long-term and focuses on the treatment process. It also focuses on emotions, deepens and develops the interaction between the children, addresses and analyzes the dynamics between the group members. According to the treatment, discussions are conducted with parents and teachers in order to help the child apply the social skills they acquired in the group in other settings. The limited number of children in the sessions (5–6) makes it possible to give each group member the necessary treatment and attention. Moreover, a study was conducted to confirm the treatment.

The results of the study are encouraging, and they indicate the need for additional studies to validate the “*Ensemble*” treatment. We suggest progressing to a full RCT study in which “*Ensemble*” groups and other, non-music therapy groups are randomized and compared after a year of treatment. We recommend that such a study repeat the approach of the current study in taking dependent measures both from within the group setting and from other daily contexts. This will increase the validity of the results and point at a possible generalization between work within the group sessions and what happens later, in daily situations. 

The positive results of this study raise the possibility of using the “*Ensemble*” treatment for different populations, although it is important to accompany each case with a study that examines whether the treatment is indeed suitable for it. First, one can try to use the treatment for children of different ages. The treatment is flexible and can be adapted to work with primary school children and even adolescents. Second, the treatment may be adapted to work with children with different developmental delays, such as verbal, communicative, or cognitive delays. The treatment will need to be adapted to the population and an accompanying study will examine its effectiveness in treating each of the populations.

This study has several research limitations. First, the study is based on a quasi-experimental design that does not include a control group. This means that there are alternative explanations for the results such as the fact the children might spontaneously improve their social skills as the year passes, and that the improvement was not necessarily the result of the “*Ensemble*” treatment. However, we selected this design to pilot test whether there is a general trend of improvement in social skills but now that the trend was identified, it is possible to continue research with a randomized control group. Second, we did not use a standardized tool to assess children’s social behaviors. This should be considered in further studies. Third, no fathers participated in this study, thus not taking into account their potentially different angle. We allowed all of the parents to participate in the study, and the natural tendency was for the mothers to step forward. Possibly, in future studies, we will give more incentive to fathers, or better even, to both parents to take part in the study and to obtain more accurate feedback on the participating children. Finally, the study was based on a limited number of children. In future studies we recommend to test the “*Ensemble*” treatment on a larger number of participants, enabling better generizability. A larger scale study will also allow to examine whether there are special features in the “*Ensemble*” treatment that address special behaviors and whether children with different disabilities are impacted from the “*Ensemble*” treatment in different ways and to different extents. 

## 5. Conclusions

In this study we conducted an initial examination of the “*Ensemble*” treatment and found that it improved children’s social skills both within the confines of the group and in other social contexts, as reported by the children’s mothers (at home) and KTs (in the kindergarten). The results were positive regardless of the diverse group composition, which indicates the “*Ensemble*” treatment’s potential with various clinical populations. These preliminary results encourage further study of the effectiveness of the “*Ensemble*” treatment, using more rigorous research designs that include comparison to control groups. Further research should also aim to better understand how to improve all social behaviors listed in the treatment and how to encourage the involvement of both parents in intake and feedback interviews.

## Figures and Tables

**Table 1:** A comparison of number of occurrences of social behaviors between first and last sessions, and between 3 first and 3 last sessions (*n* = 22).

Behaviours	First Session	Last Session	t-Value (Effect Size ^a^)	First 3 Sessions	Last 3 Sessions	t-Value (Effect Size ^a^)
Initiating contact with a friend	0.95 (1.71)	7.18 (4.84)	−5.84 (1.3) ***	3.29 (2.16)	7.18 (4.84)	−4.41 (0.9) ***
Answering a friend	0.16 (0.64)	5.36 (4.11)	−5.99 (1.3) ***	2.47 (1.73)	4.68 (3.53)	−3.47 (0.7) **
Initiating contact with the therapist	1.65 (1.88)	4.36 (3.36)	−3.05 (0.6) **	1.65 (1.53)	2.66 (1.93)	−2.87 (0.6) **
Sharing	4.29 (4.83)	12.36 (5.85)	−6.82 (1.4) ***	6.68 (3.93)	11.57 (5.21)	−5.47 (1.1) ***
Participating in group activities	15.09 (5.99)	23.31 (8.23)	−3.17 (0.7) **	15.26 (4.07)	19.5 (3.96)	−2.69 (0.6) *
Making and maintaining eye contact	6.04 (5.74)	16.68 (6.40)	−6.94 (1.5) ***	8.44 (4.31)	13.86 (5.34)	−5.67 (1.2) ***
Sharing emotions with others	0.05 (0.21)	1.40 (1.33)	−4.57 (1.0) ***	0.02 (0.07)	0.66 (0.62)	−4.83 (1.0) ***
Being considerate of a friend	0.36 (0.90)	1.27 (2.43)	−1.65	0.57 (0.64)	0.83 (1.16)	−1.08
Helping a friend	0.05 (0.21)	0 (0)	1	0.08 (0.17)	0.03 (0.10)	1.15
Reaching a compromise	0.00 (000)	0.36 (0.66)	−2.59 (0.5) *	0.04 (0.11)	0.17 (0.26)	−2.23 (0.5) *
Providing solutions to social problems	0.23 (0.51)	0 (0)	2.1 (0.4) *	0.22 (0.30)	0.015 (0.07)	3.16 (0.7) **
Delaying gratification ^b^	1.50 (2.70)	0 (0)	2.6 (0.5) *	0.60 (2.7)	0.13 (0.31)	2.19 (0.5) *

* *p* < 0.05 ** *p* < 0.01 *** *p* < 0.001; ^a^ According to Cohen’s d with Hedge’s correction for samples smaller than 50; ^b^ We counted the number of times children did not succeed in delaying their gratification. Therefore, lower frequency for this variable reflects improvement.

**Table 2:** (**a**) Summary of the difficulties of Group 1 children. (**b**) Summary of the difficulties of Group 2 children. (**c**) Summary of the difficulties of Group 3 children. (**d**) Summary of the difficulties of Group 4 children.

(**a**)														
**Group 1**	**Roi**		**Yan**		**Arthur**		**Omer**				**Leon**		**Itai**	
	**M**	**KT**	**M**	**KT**	**M**	**KT**	**M**		**KT**		**M**	**KT**	**M**	**KT**
Emotional	<	<	<	<	<	<	<		<		<	<	<	<
Social	<	<	=		<	<	<		<		<	<	<	<
Behavioral		<	<	<			<		<					
Speech					<						<	<	<	<
Attention						=								
(**b**)														
**Group 2**	**Avi**		**Nir**		**Shai**		**Omri**				**Itzhak**			
	**M**	**KT**	**M**	**KT**	**M**	**KT**	**M**		**KT**		**M**		**KT**	
Emotional	<		<	<	<		<							
Social	<	<	<	<	<	<	<		<		<		<	
Behavioral														
Speech					<									
Attention	<	<			<	<			<					
(**c**)														
**Group 3**	**Nirit**		**Tohar**		**Michael**		**Shir**		**Ethan**		**Avner**		**Liron**	
	**M**	**KT**	**M**	**KT**	**M**	**KT**	**M**	**KT**	**M**	**KT**	**M**	**KT**	**M**	**KT**
Emotional	<	<	<				<	<	<	=	<	<		
Social		<	<	<	<	<	<	<	<	<	<		<	<
Behavioral					=	=								<
Speech														
Attention					=	<								
(**d**)														
**Group 4**	**Yoav**		**Natali**		**Dani**		**Shlomi**				**Karin**		**Harel**	
	**M**	**KT**	**M**	**KT**	**M**	**KT**	**M**		**KT**		**M**	**KT**	**M**	**KT**
Emotional		<	=		<	<					=		<	<
Social	=	=	=		<	<	<				<	<	<	<
Behavioral		=			<				<					
Speech														
Attention														

Note: < improvement, > regression, = no change, empty cell—not applicable.

**Table 3:** A comparison between different treatments for enhancing social skills.

	IMP	Step-by-Step	MIT	MTSSA	Ensemble
Purpose of the treatment	Improving particular social skills	Improving particular social skills	Improving particular social skills	Improving particular social skills	Improving diverse social skills
Level of construction	Spontaneous, no guidance to facilitator	Very structured	Very structured	Structured and spontaneous	Structured and spontaneous
Target population	Developmental delays, emphasis on cognitive delay	Broad spectrum of difficulties	Autistic children and their parents	Broad spectrum of difficulties	Broad spectrum of difficulties
Mediums used	Music	Music	Music	Music	Combination of mediums
Process	No reference to the process	Process is built gradually from specific to general	Process is built gradually from specific to general	No reference to the process	Process is built gradually from specific to general
Emotions	No reference to emotions	No reference to emotions	No reference to emotions	No reference to emotions	Based on work on emotions
Group dynamics	No reference to group dynamics	No reference to group dynamics	No reference to group dynamics	No reference to group dynamics	Group dynamics are constantly monitored
Parents and teachers	No connection	No connection	Guidance for parents is provided	Built-in sharing with parents and teachers	Built-in sharing with parents and teachers
Group size	12 children	Not mentioned	1 child-parent dyad	4–7 children selected by teacher and music therapist	4–6 children selected by teacher and music therapist
Research	Research was conducted	Research was conducted	Research was conducted	Research was conducted	Research was conducted

## Data Availability

Not applicable.
